# Evaluation of the Factor Structure and Psychometric Properties of the German Version of the Clinical Perfectionism Questionnaire: The CPQ-D

**DOI:** 10.32872/cpe.3623

**Published:** 2021-06-18

**Authors:** Isabel Roth, Barbara Cludius, Sarah J. Egan, Karina Limburg

**Affiliations:** a Department of Psychology, LMU Munich, Munich, Germany; b School of Psychology and Speech Pathology, Curtin University, Perth, Australia; Philipps-University of Marburg, Marburg, Germany

**Keywords:** perfectionism, Clinical Perfectionism Questionnaire, German version, validity, factor analysis

## Abstract

**Background:**

The aim was to create a German version of the Clinical Perfectionism Questionnaire (CPQ-D) and to test its factor structure, reliability, and validity in a non-clinical population.

**Method:**

We recruited N = 432 participants via an online panel. The factor structure of CPQ-D was examined. The convergent, discriminative, and incremental validity was assessed in relation to the Frost Multidimensional Perfectionism Scale (FMPS) and the Positive and Negative Affect Schedule (PANAS).

**Results:**

Exploratory factor analysis resulted in two factors. Factor 1 represented the over evaluation of striving and Factor 2 was associated to concern over mistakes. Internal consistency was acceptable with ω = .81 for the total score, ω = .77 for Factor 1, and ω = .73 for Factor 2. Convergent, discriminative, and incremental validity was demonstrated. Important to note, Item 12 should be used with caution since it showed low communality and a low item-total correlation and should therefore be further evaluated in future research.

**Conclusion:**

The results indicate that the German translated version of the CPQ has acceptable internal consistency, convergent, discriminative and incremental validity. Future research should test the CPQ-D scale further in clinical and non-clinical populations and assess a broader variety of scales to determine validity of the scale.

## Highlights

- A German translation of the CPQ was tested and validated in a large community sample.
- The factor structure equals the English version, revealing two factors of clinical perfectionism.
- The CPQ-D proved to be a reliable and valid measure in a non-clinical sample.

Perfectionism is the tendency to set very high standards and to critically evaluate one’s own behaviour (Frost, Marten, Lahart, & Rosenblate, 1990). The construct of perfectionism is usually defined as multidimensional and mostly assessed with two Multidimensional Perfectionism Scales (FMPS; Frost et al., 1990; HMPS; Hewitt & Flett, 1991). Factor analyses of the two scales have consistently resulted in two factors: perfectionistic strivings and perfectionistic concerns (Stöber & Otto, 2006). Perfectionistic strivings refer to striving for high standards and perfectionistic concerns refer to concerns over mistakes and the belief others hold high standards of the individual. Recent meta-analytic evidence has demonstrated that both dimensions of perfectionism are linked to psychopathology, particularly eating disorders, but also depression, anxiety and obsessive-compulsive disorder (Limburg, Watson, Hagger, & Egan, 2017). In order to focus on the clinically relevant aspects of perfectionism, Shafran, Cooper, and Fairburn (2002) proposed a model of clinical perfectionism, defined as an overdependence of self-evaluation on meeting personally demanding, self-imposed standards, despite adverse consequences (Shafran et al., 2002). Thus, the multidimensional construct of perfectionism (including perfectionistic strivings and concerns) differs from clinical perfectionism as the definition of clinical perfectionism puts a central emphasis on self-worth being dependent on meeting high standards. This emphasis is not present in the definition of perfectionistic strivings and concerns. Shafran and colleagues (2002) developed a model which outlines a range of cognitive and behavioural processes which maintain clinical perfectionism. Based on the clinical perfectionism model (Shafran et al., 2002) cognitive behaviour therapy (CBT) interventions were developed to target clinical perfectionism as a transdiagnostic process which is a predisposing and maintaining process in a range of psychological disorders (Egan, Wade, & Shafran, 2011). CBT for perfectionism has been demonstrated to result in transdiagnostic reductions in anxiety, depression and eating disorders (Suh, Sohn, Kim, & Lee, 2019). This approach to treat clinical perfectionism across disorders is in line with the current approach of process-based treatment (Hofmann & Hayes, 2019). In order to evaluate treatment efficacy, it is crucial to have a psychometrically sound scale assessing clinical perfectionism.

Therefore, Fairburn, Cooper, and Shafran (2003) developed the Clinical Perfectionism Questionnaire (CPQ), consisting of 12 items that assess clinical perfectionism in the previous month. Several studies have examined the validity and reliability of the CPQ. Chang and Sanna (2012) found the CPQ was positively correlated with depression and anxiety, indicating convergent validity. The CPQ further accounted for additional variance in depression and anxiety beyond the HMPS (Hewitt & Flett, 1991), which demonstrated incremental validity (Chang & Sanna, 2012). Dickie, Surgenor, Wilson, and McDowall (2012) tested the CPQ in a non-clinical sample. They excluded Items 7 (“Have you judged yourself on the basis of your ability to achieve high standards?”) and 8 (“Have you done just enough to get by?”) due to low or negative correlations with all other items and low item-total correlations. A factor analysis of the remaining ten items resulted in two factors representing personal standards and concerns about failure with acceptable reliability (α = .71 for both factors; Dickie et al., 2012). Similar conclusions were drawn by Stöber and Damian (2014) who also excluded Items 7 and 8 because of low correlations and crossloadings on the two factors they found. Convergent validity was demonstrated by positive correlations with other perfectionism measures (Stöber & Damian, 2014). Egan and colleagues (2016) tested the psychometric properties of the CPQ including all 12 items in both a clinical eating disorder and community sample. Their factor analysis also resulted in two factors representing similar constructs as previous studies. Factor 1 comprised the overevaluation of striving, and convergent validity was indicated by a significant positive correlation (*r* = .64) with the FMPS subscale *personal standards.* Factor 2 was related to reacting to perceived failure, and convergent validity was demonstrated with self-criticism indicated by substantial and significant positive correlations with the FMPS subscales *concern over mistakes* (*r* = .61) and *doubts about actions* (*r* = .56). Further indicating convergent validity, the second factor of the CPQ was correlated with the negative affect subscale of the *Positive and Negative Affect Schedule* (PANAS; Watson, Clark, & Tellegen, 1988). Discriminant validity of the CPQ was shown because it could reliably discriminate between both participants with high and low negative affect as well as between the eating disorder sample and healthy controls. In terms of incremental validity, the FMPS accounted for 23% of variance while the CPQ accounted for an additional 11% of variance in the PANAS-NA scores (Egan et al., 2016). Prior and colleagues (2018) also found in a clinical eating disorder sample a two factor structure using a bifactor approach, comprising of overevaluation of striving and concern over mistakes, in a 10 item version of the CPQ excluding the two items found in previous research to be problematic. Due to the focus of the CPQ on clinical aspects of perfectionism relevant to treatment, the aim of this study was to develop a German version of the scale in order to extend access to and distribution of the CPQ. This is important in further evaluating the efficacy of CBT for perfectionism in German speaking areas in clinical practice and research. In the present study a German translation of the CPQ was developed and tested within a community sample in order to explore the factor structure and psychometric properties of the scale. Since this is the first study on a German version, we used all 12 items instead of the reduced set of 10 items. We hypothesized that the German version (CPQ-D) would consist of two factors with a similar structure to the English version found in previous research (Egan et al., 2016; Prior et al., 2018) and that convergent, discriminant, and incremental validity would be demonstrated.

## Materials and Methods

### Sample

We used a community sample and recruited participants via the online panel PsyWeb (https://psyweb.uni-muenster.de). Inclusion criteria were age above 18 years and self-reported good German language abilities. Since sample sizes of *N* = 200-300 are regarded suitable for a factor analysis even with lower communalities of the items, we aimed to recruit a minimum sample of *N* = 250 (Bühner, 2011).

### Measures

To create the German version of the CPQ (CPQ-D), the original version of the CPQ was first translated into German by the first author, then translated back to English and compared to the original version by the senior author. Finally, a few linguistic changes were made by the first and the senior author. The original CPQ (Fairburn et al., 2003) is a self-report measure that assesses the core elements of clinical perfectionism (see Table 2). The 12 items, of which Items 2 and 8 are reverse-scored, are rated based on participants’ past 28 days on a 4-point Likert scale from 1 (*not at all*) to 4 (*all the time*). Total scores therefore range from 12 to 48 and a higher score indicates a higher level of clinical perfectionism.

The German version of the Frost Multidimensional Perfectionism Scale (FMPS; Frost et al., 1990; Stöber, 1995) was used to assess multidimensional perfectionism with six subscales: personal standards (PS), concern over mistakes (CM), doubts about actions (DA), parental expectations (PE), parental criticism (PC), and organisation (O) and a sum score. The FMPS-D was chosen because its subscales personal standards and concern over mistakes are close to the definition of clinical perfectionism (Egan et al., 2016; Shafran et al., 2002). It consists of 35 items rated on 5-point Likert scales from 1 (*strongly disagree*) to 5 (*strongly agree*). Following recommendations of Dunn, Baguley, and Brunsden (2014), McDonald’s ω (McDonald, 1999) was used instead of Cronbach’s α to examine internal consistency. For the FMPS it was acceptable with ω = .92 for concern over mistakes, ω = .84 for personal standards and ω = .76 for doubts about actions. The FMPS score in our study comprised the subscales personal standards, doubts about actions, and concern over mistakes, following previous research examining the validity of the CPQ (Egan et al., 2016).

We used the German version of the Positive and Negative Affect Schedule (PANAS; Krohne, Egloff, Kohlmann, & Tausch, 1996; Watson et al., 1988) to measure positive and negative affect over the past 28 days. The scale contains ten words describing pleasant and ten words describing unpleasant emotions, representing the subscales positive affect (PA) and negative affect (NA), respectively. Participants rate to what extent they had experienced each of the 20 emotions during the past weeks on a 5-point scale. The PANAS is valid (Krohne et al., 1996) and in the present sample the internal consistency for the positive affect scale (PANAS-PA) was ω = .90 and for the negative affect scale (PANAS-NA) was ω = .89.

### Procedure

The study was approved by the ethics committee of the faculty for psychology and educational science at the Ludwig-Maximilians University Munich. Participants provided informed consent and there was no identifying data. The online survey started with a short introduction after which participants were asked to complete the CPQ-D, the FMPS-D and the PANAS. Finally, personal feedback regarding individual results on the FMPS-D was provided.

### Statistical Analyses

The free software R, version 3.5.1 (R Core Team, 2019), was used for all statistical analyses. The following additional packages were necessary for the analyses: GPArotation (Bernaards & Jennrich, 2005), boot (Canty & Ripley, 2017), semPlot (Epskamp, 2019), QuantPsych (Fletcher, 2012), Polycor (Fox, 2016), Car (Fox & Weisberg, 2019), Hmisc (Harrell, 2019), MBESS (Kelley, 2019), ggm (Marchetti et al., 2015), Foreign (R Core Team, 2018), Psych (Revelle, 2018), Corpcor (Schafer et al., 2017), effsize (Torchiano, 2018), ggplot2 (Wickham, 2016). Significance level for all tests was α=.05. After calculating descriptive statistics, Bartlett’s test was used to test for sphericity and Kaiser-Meyer-Olkin test was applied to examine sampling adequacy. Further, inter-item-correlations were calculated to investigate whether all 12 items could be included in the exploratory factor analysis (EFA). Afterwards and based on the results of the preceding tests, an EFA was conducted for the CPQ-D. The number of factors was determined with a scree plot and a parallel analysis. In the parallel analysis the eigenvalues of the empirical data were compared against the 95^th^ percentile of eigenvalues generated from 1000 simulated analyses, corresponding in size and number of items. To not risk keeping too many or irrelevant factors, the conservative approach of using only the 95^th^ percentile of the simulated eigenvalues was applied. Factors with actual eigenvalues greater than those simulated eigenvalues were maintained (Hayton, Allen, & Scarpello, 2004).

Again, McDonald’s ω was used instead of Cronbach’s α to examine internal consistency of the factors (Dunn et al., 2014; McDonald, 1999). To test convergent and discriminative validity, correlations between the measures were calculated. Substantial and significant positive correlations between the CPQ-D, the FMPS-D, and PANAS-NA were considered evidence for convergent validity. In terms of discriminant validity, small positive and/or negative correlations were expected between the CPQ-D and PANAS-PA. Correlation coefficients were interpreted according to the rule of thumb by Cohen (1988), with 0.1≤|*r*|< 0.3 indicating small, 0.3≤|*r*|< 0.5 indicating moderate, and |*r*|> 0.5 indicating high correlations. To further test discriminant validity, we conducted *t*-tests to examine if participants with low negative affect differed from those with high negative affect in their CPQ-D scores. Effect sizes were assessed with Cohen’s *d* and interpreted as small if 0.2≤|*d*|< 0.5, medium if 0.5≤|*d*|< 0.8, and high if |*d*|> 0.8 (Cohen, 1988). Finally, a hierarchical linear regression analysis predicting the PANAS-NA score with the FMPS-D and CPQ-D scores as independent variables was conducted to check for incremental validity.

## Results

### Participants

We collected data from 439 participants. Data screening resulted in the exclusion of three datasets due to missing consent, two were excluded due to invalid age information, one due to voluntary withdrawal, and one due to insufficient knowledge of the German language. The final sample consisted of *N* = 432 participants. Descriptive data of the sample along with means and standard deviations for the CPQ-D, FMPS-D, and PANAS are presented in Table 1. The mean CPQ-D total was *M* = 26.50 (*SD* = 5.70).

**Table 1:** Sample Characteristics, N = 432

Variable	*M* (*SD*) or *n* (%)
**Age (years), *M* (*SD*)**	49.53 (15.00)
**Female, *n* (%)**	251 (58.10)
Education, *n* (%)	
9th grade or less	19 (4.40)
10th grade	62 (14.35)
High school graduate	102 (23.61)
University graduate	243 (56.25)
Other degree	6 (1.39)
**Psychological diagnosis, *n* (%)**	137 (31.71)
Psychotherapeutic and/or psychiatric treatment, *n* (%)	
Yes, currently, *n* (%)	61 (14.12)
Yes, formerly, *n* (%)	162 (37.50)
Never, *n* (%)	233 (53.94)
CPQ-D total, *M* (*SD*)	26.50 (5.70)
Factor 1, *M* (*SD*)	18.21 (4.17)
Factor 2, *M* (*SD*)	8.29 (2.41)
FMPS-D total, *M* (*SD*)	98.84 (21.24)
Personal Standards, *M* (*SD*)	21.64 (5.50)
Doubts about Actions, *M* (*SD*)	9.99 (3.82)
Concern over Mistakes, *M* (*SD*)	22.11 (8.43)
Parental Expectations, *M* (*SD*)	11.98 (5.11)
Parental Criticism, *M* (*SD*)	10.11 (4.61)
Organisation, *M* (*SD*)	23.02 (4.48)
**PANAS-NA, *M* (*SD*)**	20.61 (7.74)
**PANAS-PA, *M* (*SD*)**	31.91 (7.39)

*Note.* CPQ-D = Clinical Perfectionism Questionnaire, German version; FMPS-D = Frost Multidimensional Perfectionism Scale; PANAS-NA = Positive and Negative Affect Schedule, Negative Affect subscale; PANAS-PA = Positive and Negative Affect Schedule, Positive Affect subscale.

### Factor Structure and Internal Consistency

Inter-item correlations were mostly moderate, only Items 8 and 12 had small correlations to other items (*r* < .30). The same items had small item-total correlations of *r* = .19 for Item 8 and *r* = .20 for Item 12. Due to results of Bartlett’s test, χ^2^(66) = 1253.53, *p* < .001, and KMO test (MSA = .85) and since inter-item correlations were significant for all items, we decided to run the factor analysis for the complete set of items instead of excluding Items 8 and 12. An exploratory factor analysis using maximum likelihood estimation with promax rotation resulted in two factors with simple structure. Two factors were assumed based on the scree plot and parallel analysis. Of note, the eigenvalue rule was not fulfilled with only one factor having an eigenvalue greater than one, but the eigenvalue criterion has been marked as too strict (Jolliffe, 1972). Eight items loaded on Factor 1 and four items on Factor 2. Factor 1 explained 20% and Factor 2 accounted for 15% of variance, factors were moderately correlated with *r* = .49. The factor structure along with communalities of the items is depicted in Table 2. Internal consistency was ω = .81 for the total score, ω = .77 for Factor 1, and ω = .73 for Factor 2.

**Table 2:** Promax-Rotated Factor Structure of the CPQ-D

Item No.	Item CPQ	Item CPQ-D	F1	F2	*h* ^2^
1	Have you pushed yourself really hard to meet your goals?	Haben Sie sich sehr angestrengt, um Ihre Ziele zu erreichen?	**.48**	.08	.27
3	Have you been told that your standards are too high?	Wurde Ihnen gesagt, dass Ihre Ansprüche zu hoch sind?	**.53**	.12	.36
6	Have you raised your standards because you thought they were too easy?	Haben Sie Ihre Ansprüche erhöht, weil Sie dachten sie seien zu leicht zu erreichen?	**.46**	.00	.21
7	Have you judged yourself on the basis of your ability to achieve high standards?	Haben Sie sich selbst anhand dessen beurteilt, ob Sie hohe Ansprüche erreichen können?	**.58**	.23	.51
8	Have you done just enough to get by? (R)	Haben Sie geradeso genug getan, um über die Runden zu kommen? (R)	**.42**	-.23	.14
9	Have you repeatedly checked how well you are doing at meeting your standards (for example, by comparing your performance with that of others)?	Haben Sie wiederholt überprüft, wie gut Sie sich darin schlagen, hohe Ansprüche zu erreichen (zum Beispiel, indem Sie Ihre Leistung mit der anderer verglichen)?	**.51**	.20	.39
10	Do you think that other people would have thought of you as a “perfectionist”?	Glauben Sie, dass andere Leute Sie als „Perfektionist/in“ bezeichnen würden?	**.62**	-.14	.32
11	Have you kept trying to meet your standards, even if this has meant that you have missed out on things?	Haben Sie auch dann versucht, Ihre Ansprüche zu erreichen, wenn Sie dadurch andere Dinge versäumt haben?	**.56**	.05	.34
2	Have you tended to focus on what you have achieved, rather than on what you have not achieved? (R)	Haben Sie sich eher darauf fokussiert was Sie erreicht haben, als darauf, was Sie nicht erreicht haben? (R)	-.03	**.45**	.19
4	Have you felt a failure as a person because you have not succeeded at meeting your goals?	Haben Sie sich wie ein/e Versager/in gefühlt, wenn es Ihnen nicht gelang, Ihre Ziele zu erreichen?	.14	**.76**	.70
3	Have you been afraid that you might not reach your standards?	Hatten Sie Angst, dass Sie Ihre Ziele möglicherweise nicht erreichen könnten?	.14	**.71**	.61
12	Have you avoided any tests of your performance (at meeting your goals) in case you failed?	Haben Sie jede Art der Überprüfung Ihrer Leistung vermieden, weil Sie bei der Erreichung Ihrer Ziele versagt haben könnten?	-.03	**.35**	.11

*Note*. F1 = loadings on Factor 1; F2 = loadings on Factor 2; h^2^ = communality; (R) = reverse-coded, loadings > 0.3 are printed in bold.

Reprint of original items with courtesy of Roz Shafran.

### Construct Validity

Pearson’s correlations between the measures are seen in Table 3.

**Table 3:** Pearson Correlations and Partial Correlations of the Scales

Scale	CPQ-D total	Factor 1	F1.F2	Factor 2	F2.F1
FMPS-D total	.68*** [.63, .73]				
Personal Standards	.60*** [.54, .66]	.66*** [.60, .71]	.62***	.29*** [.20, .38]	-.02
Concern over Mistakes	.67*** [.62, .72]	.55*** [.48, .61]	.37***	.61*** [.59, .70]	.53***
Doubts about Actions	.51*** [.44, .58]	.35*** [.27, .43]	.09	.65*** [.55, .67]	.54***
PANAS-NA	.55*** [.48, .61]	.40*** [.32, .48]	.17***	.61*** [.55, .66]	.52***
PANAS-PA	-.11* [-.21, -.02]	.07 [-.03, .16]		-.39*** [-.46, -.30]	

*Note.* CPQ-D = Clinical Perfectionism Questionnaire, German version; FMPS = Frost Multidimensional Perfectionism Scale; PANAS-NA = Positive and Negative Affect Schedule, Negative Affect subscale; PANAS-PA = Positive and Negative Affect Schedule, Positive Affect subscale. Values in brackets depict the 95% CI for the respective Pearson correlation coefficient. F1.F2 = partial correlation of Factor 1 controlled for Factor 2, F2.F1 = partial correlation of Factor 2 controlled for Factor 1.

*p < .05. **p < .01. ***p < .001.

#### Convergent Validity

The CPQ-D total was highly correlated with the FMPS-D total and the relevant subscales personal standards, concern over mistakes, and doubts about actions, and with PANAS-NA. Factor 1 correlated with personal standards, but also concern over mistakes. When controlling for overlap with Factor 2, the relationship was only moderate. Factor 2 correlated highly with concern over mistakes, doubts about actions, and PANAS-NA and the relationship remained when controlling for Factor 1. Hence, the CPQ-D demonstrated convergent validity.

#### Discriminative Validity

As expected, correlations between CPQ-D and both factors and PANAS-PA were small to negative. Following Egan and colleagues (2016), we classified participants with PANAS-NA scores of > 25 (75^th^ percentile) as “high” (*n* = 114) and those with scores < 15 (25^th^ percentile) as “low” (*n* = 133). An independent samples t-test revealed that those with higher PANAS-NA scores had significantly higher scores on CPQ-D total than those with low PANAS-NA scores (“high” PANAS-NA group: *M* = 31.18, *SD* = 5.42; “low” PANAS-NA group: *M* = 23.14, *SD* = 4.44; *t*(218.51) = 12.61, *p* < .001). Cohen’s *d* was large with *d =* 1.63 (95% CI [1.34, 1.92]). Similar findings were evident for Factor 1 and Factor 2 (Factor 1: “high” PANAS-NA group: *M* = 20.75, *SD* = 4.01; “low” PANAS-NA group: *M* = 16.38, *SD* = 3.60; *t*(226.94) = 8.84, *p* < .001, *d* = 1.14, 95% CI for *d* [.87, 1.41]; Factor 2: “high” group: *M* = 10.43, *SD* = 2.26; “low” group: *M* = 6.77, *SD* = 1.62; *t*(201.23) = 14.40, *p* < .001, *d* = 1.88, 95% CI for *d* [1.58, 2.19]).

#### Incremental Validity

A multiple hierarchical linear regression model showed that the FMPS-D accounted for 23.6% of variance in PANAS-NA (*p* < .001) and that the CPQ-D accounted for an additional 11% of variance (*p* < .001). Upon inclusion of the CPQ-D total in the regression model, the predictive value of the FMPS-D reduced from β = .49 to β = .21, which could be due to the strong correlation of both variables (*r* = .68). The variance inflation factor of 1.86 confirmed that there was no multicollinearity between the predictors. Hence, in the final model including FMPS-D and CPQ-D, the latter was a stronger predictor for negative affect than the FMPS-D.

## Discussion

Consistent with previous studies on the original version of the CPQ, the CPQ-D consists of two factors, with the same eight items loading on Factor 1 as the respective items in the English version and the same four items loading on Factor 2 (Dickie et al., 2012; Egan et al., 2016; Stöber & Damian, 2014). The values of the loadings of the single items differ slightly between all sighted analyses, but never by more than 0.15 between the German and the English version (Egan et al., 2016). Similar to previous studies Factor 1 represents primarily the over evaluation of striving whereas Factor 2 assesses concern over mistakes (Egan et al., 2016; Prior et al., 2018). Unlike the English version, the German version contains no cross loadings greater than 0.3 on both factors, which suggests that the German translation might discriminate more precisely between the two factors. Internal consistency of the factors and the total score were acceptable. The amount of variance explained by both factors was 35%, a very low proportion considering recommendations that at least 60% of variance should be explained (Hair et al., 2013). Previous studies found diverging amounts of variance explained, with 47.9% (Dickie et al., 2012), 45.9% (Stöber & Damian, 2014), and 79% (Egan et al., 2016). The low proportion we found could indicate that there is a third latent variable behind the construct of clinical perfectionism that could not be covered by the items. Alternatively, formulation of the translated items may not be adequate so that they cannot sufficiently assess the two latent variables. Prior and colleagues (2018) argued that a single, latent construct of clinical perfectionism could also explain the structure of the CPQ in a clinical eating disorder sample, and it is possible that a unidimensional structure may be worth further investigating in future research.

A noteworthy finding was that Items 8 and 12 had both low communalities, indicating small associations with both factors, and low item-total correlations, indicating that these items insufficiently represent the total scale. Findings for Item 8 (“Have you done just enough to get by?”) can be interpreted in accordance with previous research finding this reverse scored item problematic (Dickie et al., 2012; Prior et al., 2018). This is supported by Item 8 having relatively high loadings with opposite items on both factors, which means that participants with a high score on Factor 1 (over evaluation of striving) seem to interpret Item 8 in an opposite way to participants with high scores on Factor 2 (concern over mistakes). This is likely due to the item being reverse scored and participants were reading the item incorrectly assuming it was similar to other items. Future research on the German CPQ should examine the 12-item version and a 10-item version of the scale with the reverse scored items removed. Item 12 (“Have you avoided any test of your performance (at meeting your goals) in case you failed?”) was not problematic in studies on the English version. They found that Item 12 loaded on Factor 2 between .37 and .71 and had corrected item total correlations (CITC) of .24 or higher. In the German version the loading on Factor 2 was slightly smaller, but more problematic were the low CITC of .20 and the low communality of .11. This indicates that Item 12 does not represent Factor 2 well and does not contribute much to assessing the construct of clinical perfectionism. One reason could be that the German translation of Item 12 may have been too complicated to be easily understood by participants. Furthermore, avoidance of performance tests could be associated with other factors than perfectionism, for example test anxiety, a lack of motivation to be tested, or simply having no test situations available in everyday life. Future research on the CPQ-D should address this issue because the original content of Item 12 (testing and evaluating one’s performance) is an important part of the definition of clinical perfectionism.

In terms of validity, our results provided evidence for convergent validity, discriminative validity, and incremental validity. Convergent validity was demonstrated by high correlations with the FMPS-D and the negative affect subscale of the PANAS. Factor 1 correlated highly with FMPS-D subscales assessing the setting and evaluation of strivings while Factor 2 correlated with scales measuring concerns about mistakes, concerns regarding meeting personal standards, and negative emotions. This supports the interpretation of Factor 1 representing perfectionistic strivings and Factor 2 assessing emotional consequences of failure. Discriminative validity was shown by low correlations with the positive affect subscale of the PANAS and by the finding that the CPQ-D could discriminate well between participants with high vs. low negative affect. Finally, the CPQ-D explained variance in negative affect beyond the proportion explained by the FMPS-D, demonstrating incremental validity.

### Strengths and Limitations

Considering that we had similar findings compared to previous studies on the English version of the CPQ in terms of factor structure and construct validity, it seems like translation of the measure was successful. Also, it shows a simple structure which ensures interpretability. Another strength is that we tested the CPQ-D not only in a student sample, but in a community sample, of which nearly a third of the participants had a self-reported diagnosed psychological disorder and 14% reported to be in psychotherapeutic and/or psychiatric treatment, indicating some generalisability towards clinical samples.

However, there were some limitations. First, the community sample was recruited using an online panel. This method only reaches certain target groups. Participants in our sample were on average 49.53 years old and highly educated, which decreases generalisability of our results (e.g., our results may not apply to younger or people with lower education). Future research should consider using test theory to explore item-person fit.

Second, we did not assess the number of specific psychological disorders, although it would be interesting to know whether there are diverging results for different disorders. Third, we used a limited number of measures to assess construct and incremental validity. Other measures assessing perfectionism and further constructs (e.g., depression, anxiety, eating disorder symptoms, general well-being, personality traits) would have been valuable to examine validity more comprehensively. Fourth, regarding translation of the measure, it would have been worthwhile to have the German version translated back to English by several people and to have the German scale evaluated by several clinicians. Moreover, it should be considered in future research to use a cognitive interview to validate the German translation. Finally, there are no “clinical” cut-offs or severity ranges for the CPQ. Instead, clinicians and researchers currently interpret the score on the basis of higher scores indicating greater clinical perfectionism. It would be useful for future research to determine severity ranges (e.g., mild, moderate, severe) to further enhance the clinical and research application of the scale.

### Conclusion

Overall, we found evidence for the reliability and validity of the CPQ-D, the factor structure is the same as in the English version (Dickie et al., 2012; Egan et al., 2016; Prior et al., 2018; Stöber & Damian, 2014). Therefore, the CPQ-D can be used in a similar way to the English version. It would be useful for future research to examine if there are differences between clinical perfectionism across countries, for example, between the United Kingdom (UK) where the CPQ was developed, and Germany. To date, cultural differences in the definition and perception of perfectionism have been found when comparing individualistic and collectivistic cultures, for example, Caucasian and Asian samples (Nilsson, Paul, Lupini, & Tatem, 1999; Pietrabissa et al., 2020). As both Germany and the UK are individualistic cultures which share common values (Juslin, Barradas, Ovsiannikow, Limmo, & Thompson, 2016) we do not assume great cultural differences. However, future research should test this possible effect on the results. Future studies should also examine the CPQ-D in non-clinical and clinical populations in order to evaluate whether the factor structure can be replicated and whether it is possible to explain more variance of the underlying construct than in the current study. Additionally, they should use a wider variety of additional measures to test its validity. Further, future research may wish to compare the CPQ-D in its current version with a version that excludes Items 8 and 12 due to their difficult properties. In summary, the CPQ-D appears to be a valid and reliable measure to assess clinical perfectionism in a German speaking population. Hence, it has the potential to be used as an efficient measure to assess the process of clinical perfectionism within the framework of process-based CBT (Hofmann & Hayes, 2019).
